# Placental Endocrine Activity: Adaptation and Disruption of Maternal Glucose Metabolism in Pregnancy and the Influence of Fetal Sex

**DOI:** 10.3390/ijms222312722

**Published:** 2021-11-24

**Authors:** Christina Stern, Sarah Schwarz, Gerit Moser, Silvija Cvitic, Evelyn Jantscher-Krenn, Martin Gauster, Ursula Hiden

**Affiliations:** 1 Department of Obstetrics and Gynecology, Medical University of Graz, 8036 Graz, Austria; christina.stern@medunigraz.at (C.S.); sarah.schwarz@stud.medunigraz.at (S.S.); evelyn.jantscher-krenn@medunigraz.at (E.J.-K.); 2 Division of Cell Biology, Histology and Embryology, Gottfried Schatz Research Center, Medical University of Graz, 8010 Graz, Austria; gerit.moser@medunigraz.at; 3 Research Unit of Analytical Mass Spectrometry, Cell Biology and Biochemistry of Inborn Errors of Metabolism, Department of Pediatrics and Adolescent Medicine, Medical University of Graz, 8036 Graz, Austria; silvija.tokic@medunigraz.at

**Keywords:** pregnancy, placental endocrine function, glucose metabolism, insulin resistance, gestational diabetes, sex dimorphism

## Abstract

The placenta is an endocrine fetal organ, which secretes a plethora of steroid- and proteo-hormones, metabolic proteins, growth factors, and cytokines in order to adapt maternal physiology to pregnancy. Central to the growth of the fetus is the supply with nutrients, foremost with glucose. Therefore, during pregnancy, maternal insulin resistance arises, which elevates maternal blood glucose levels, and consequently ensures an adequate glucose supply for the developing fetus. At the same time, maternal β-cell mass and function increase to compensate for the higher insulin demand. These adaptations are also regulated by the endocrine function of the placenta. Excessive insulin resistance or the inability to increase insulin production accordingly disrupts physiological modulation of pregnancy mediated glucose metabolism and may cause maternal gestational diabetes (GDM). A growing body of evidence suggests that this adaptation of maternal glucose metabolism differs between pregnancies carrying a girl vs. pregnancies carrying a boy. Moreover, the risk of developing GDM differs depending on the sex of the fetus. Sex differences in placenta derived hormones and bioactive proteins, which adapt and modulate maternal glucose metabolism, are likely to contribute to this sexual dimorphism. This review provides an overview on the adaptation and maladaptation of maternal glucose metabolism by placenta-derived factors, and highlights sex differences in this regulatory network.

## 1. Modulation of Glucose Metabolism in Normal Pregnancy

In physiologic pregnancies, profound physical, hormonal and humoral changes take place, aiming to allocate essential resources of nutrition for fetal development and the increased maternal demands during pregnancy, delivery and breastfeeding. This unique period of adaptability of the female organism in human reproduction is increasingly acknowledged as a window into the future health of both mother and offspring [1]. One of the most crucial and critical metabolic adjustment is the temporal adaptation of insulin sensitivity and production [2,3]. This adaptation of maternal glucose metabolism may in turn also affect lipid metabolism and increase oxidative stress levels, particularly if the pregnancy related adaptations exceed normal glucose tolerance [1,2]. However, in this review we will focus on the adjustment of glucose metabolism in pregnancy.

### 1.1. Establishment of Insulin Resistance in Pregnancy

Throughout pregnancy, fasting glucose values continuously fall. Underlying mechanisms are not completely understood, but an increased glucose utilisation by the developing fetus and the placenta may contribute [4,5]. 

In the first trimester, the reduction of fasting blood glucose is paralleled by increasing insulin sensitivity, allowing to absorb and store large amounts of glucose as an energy resource for advanced pregnancy [6]. In ongoing pregnancy, maternal insulin sensitivity decreases and maternal insulin levels increase up to 50%, which is accompanied by a progressive rise in postprandial glucose levels and a prolonged glucose peak [4,5,7]. This phenomenon is promoted by the production of local and placental hormones, such as estrogen, progesterone, leptin, cortisol, placental lactogen and placental growth hormone [8]. Increasing maternal insulin resistance leads to a rise of maternal blood glucose concentrations, and thus facilitates glucose transport across the placenta, supporting the rapid growth of the fetus [3,6].

Glucose passively passes the placenta along the concentration gradient between the maternal and the fetal compartment. This gradient is maintained by the high basal insulin secretion of fetal β-cells, which themselves exhibit a relative glucose insensitivity, resulting in low fetal blood glucose levels [9]. In fact, fetal glucose levels are lower than maternal levels [10,11]. 

Maternal insulin resistance is physiological in pregnancy, mostly expressed at skeletal muscle and adipose tissue level. It is critically important for maintenance of maternal fuel supply to support the growing fetus, especially during the third trimester [12]. The physiologic factors underlying the decrease of insulin sensitivity are not fully elucidated. However, they seem to be associated with the metabolic effects of several hormones and bioactive proteins derived from the placenta and elevated in the maternal circulation during pregnancy. This speaks to a pivotal role of the placenta in the development of insulin resistance in pregnancy [5,12].

### 1.2. Balancing of Insulin Resistance by Expansion of β-Cell Function

Whilst the maternal organism has to meet the fetal energy demand by inducing insulin resistance, it also has to maintain maternal glucose homeostasis [3]. Animal studies have shown that during healthy pregnancy, insulin resistance results in pancreatic β-cell hyperplasia, hypertrophy and increased glucose-stimulated insulin secretion [6], counteracting an excessive glucose shift to the fetus. A balance between maternal insulin resistance and increased β-cell mass ensures optimal glucose supply to the fetus [6]. Placental derived hormones and proteins significantly contribute to the temporal adaptation of maternal β-cell mass in pregnancy with an increase in β-cell proliferation and size [13]. 

**Figure 1:**
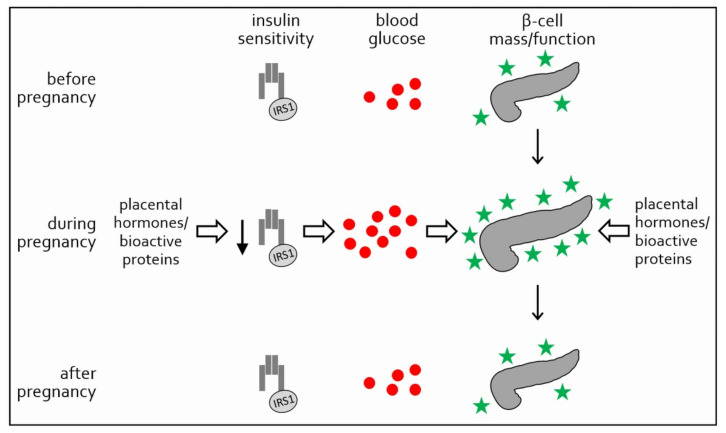
Insulin sensitivity, blood glucose and β-cell mass and function during normal pregnancy. As a consequence to insulin resistance induced by placental hormones, blood glucose rises (red circles). Due to elevated blood glucose and several placenta derived hormones and bioactive proteins, β-cells undergo hyperplasia and hypertrophy and produce higher amounts of insulin (green stars) (Modified from [6]).

Around parturition, maternal β-cell mass begins to shrink to its pre-pregnancy size [9] and within few days after delivery, also insulin sensitivity returns to pre-pregnancy levels, highlighting the role of placental hormones in altered glucose homeostasis and insulin sensitivity during pregnancy [6] (Figure 1). 

### 1.3. Outlook

This review will discuss the role of placental derived hormones and bioactive molecules in the adaptation of maternal glucose metabolism in normal pregnancy, and in the development of disturbed glucose metabolism in GDM. A special focus is put on sex dimorphism in maternal glucose metabolism in pregnancy, and the role of placenta derived molecules will be discussed.

## 2. Methodology

Publications were searched in PubMed database (https://pubmed.ncbi.nlm.nih.gov/ last accessed on 10 August 2021). For those sections dealing with sex differences, we searched with the combination ‘fetal sex’ or ‘fetal gender’ and ‘maternal insulin resistance’, ‘maternal glucose metabolism’, ‘GDM’ or the name of the corresponding hormone or signal protein. From the obtained publications, those that evaluated the metabolic parameters or signaling molecules in the maternal circulation (not amniotic fluid or umbilical cord blood) according to the sex of the fetus/neonate were selected and included. Studies that examined larger cohorts were weighted more heavily. 

## 3. Gestational Diabetes, Pathophysiology and Screening

### 3.1. Pathophysiology of GDM: Insulin Resistance and β-Cell Failure

Disruption of physiological glucose metabolism in pregnancy leads to GDM. GDM is defined as hyperglycemia first recognized in pregnancy and can be seen as consequence of an insulin supply inadequate to meet the demands for normal blood glucose regulation [14]. A comprehensive and concluding model explaining the pathophysiology of GDM is still missing, but the general insulin resistance in pregnancy was identified as the accelerator of GDM development [15]. Hyperglycemia is postulated to result from an insulin supply inadequate to meet demands of normal blood glucose regulation [14] due to β-cell dysfunction [16]. When maternal β-cells are unable to adapt to the metabolic changes accompanying pregnancy, this leads to recurrent hyperglycemic episodes manifesting in GDM [6,17]. Thus, GDM is defined as hyperglycemia first recognized in pregnancy and can be seen as consequence of an insulin supply inadequate to meet the demands for normal blood glucose regulation [14]. Both, β-cell impairment and tissue insulin resistance are critical components of the pathophysiology of GDM, eventually resulting in an inadequate regulation of glucose hemostasis during pregnancy. 

Hyperglycemia during pregnancy is consistently associated with adverse maternal and neonatal outcomes [18], and markedly contributes to health risks after birth in both mothers and babies. This leads to a further amplification of the pandemic of non-communicable diseases [15,19]. 

### 3.2. GDM Screening and Risk Factors

Diabetes is one of the most common medical disorder of pregnancy, with significant impact on the maternal-fetal dyad [20]. Globally, the prevalence of GDM is rising and the International Diabetes Federation reports that worldwide, 14% of pregnancies are complicated by GDM (https://idf.org/ last accessed on 30 July 2021). In some populations, GDM affects up to 41.9% of pregnancies [21]. 

Standard GDM screenings in ‘metabolically healthy’ women are used between gestational weeks 24-28 with several screening schemes. A two-step strategy (non-fasting 50 g glucose challenge test followed by a 1 h venous glucose test) is commonly used in the United States, and a one-step procedure (with a 2 h 75 g oral glucose tolerance test; oGTT) is recommended by the International Association of Diabetes and Pregnancy Study Group (IADPSG) [22]. The ‘IADPSG- criteria’ are broadly accepted and recommended by international health-care societies. They define the diagnosis of GDM when at least one of the thresholds is equaled or exceeded (fasting plasma glucose 5.1 mml/mol or 92 mg/dL, 1-h plasma glucose 10.0 mmol/mol or 180 mg/dL, 2-h plasma glucose 8.5 mmol/mol or 153 mg/dL) [22]. 

Criteria associated with higher risk for GDM include overweight or obese BMI (BMI ≥ 30 kg/m^2^), metabolic syndrome, pre-diabetes, polycystic ovarian syndrome, age over 35 years, family history of type 2 diabetes mellitus (T2D) and ethnicity. Excessive gestational weight gain, medical history of GDM in a previous pregnancy, previous birth of a neonate >4500 g, or multiple pregnancies, as well as lifestyle factors such as unhealthy diet before and during pregnancy, or physically inactive lifestyle are also known risk factors [6,23].

## 4. Adaptation and Disruption of Maternal Glucose Metabolism Depends on Fetal Sex

In recent years, it has become apparent that maladaptation of maternal glucose metabolism in GDM is influenced by fetal sex. Several large cohort studies revealed a higher risk to develop GDM when women were pregnant with boys [24,25,26]. Moreover, not only prevalence, but also severity of GDM seems higher in pregnancies with a male fetus. Thus, the risk for requiring insulin therapy is higher in pregnancies with boys, as well [27] as the risk for the mother to develop T2D later in life [28]. A meta-study based on data of 2.4 million women underpinned this finding by calculating a by 4% increased risk of developing GDM in pregnancies with male fetuses [29]. A more detailed analysis of glucose metabolism revealed that in mothers carrying a boy, the increase in mean adjusted blood glucose at oGTT was paralleled by a decrease in mean adjusted β-cell function [25]. 

The altered incidence of GDM between pregnancies with male vs. female fetuses points towards sex differences in the regulation of maternal glucose metabolism in normal pregnancy, as suggested by recent literature. However, results show different specific effects of sex on the modulation of glucose metabolism in different cohorts. Analyzing a cohort of 582 primarily overweight women, Walsh et al. [30] observed higher insulin resistance (HOMA-IR) in pregnancies with boys at the end of the first trimester. In a cohort of 877 women, Geng et al. [31] found higher fasting blood glucose and lower HOMA-β, a measure for β-cell function, in the second trimester of pregnancies with male fetuses. By contrast, Yamashita et al. [32] and Xiao et al. [33] observed higher maternal insulin resistance in pregnancies with female fetuses at 24-28 weeks gestation. Measuring fasting blood glucose longitudinally throughout pregnancy in a cohort including only overweight and obese women, Rafferty and colleagues observed no effect of fetal sex [34]. As the investigated cohorts differ in average BMI of the participants, in the proportion of women suffering from glucose metabolism disorders, or ethnicity, results are difficult to compare or extrapolate to the general population. Thus, more homogenous cohort studies are needed to obtain a better understanding of sex mediated differences in regulation of glucose metabolism. 

Sex differences in maternal glucose metabolism in pregnancy suggest that fetus derived signals altering maternal metabolism may differ between male and female fetuses. The placenta is a fetal organ at the interface between mother and fetus and exhibits massive endocrine activity. Signal molecules such as hormones, cytokines and growth factors modulate maternal metabolism in pregnancy. Thus, a sex dimorphism in placental release of these bioactive molecules may underlie a sex dimorphism in maternal glucose metabolism. The following sections highlight the placenta as an endocrine organ, as well as the hormones, growth factors and metabolically active proteins produced by the placenta, their effects on maternal glucose metabolism, and potential sex differences. 

## 5. The Placenta: Endocrine Organ of the Fetus

The placenta is a highly active endocrine organ, and its function is crucial for pregnancy development and adaptation of the maternal metabolism, endocrine and immune system to pregnancy. Most relevant for the endocrine activity of the placenta is the multinucleated syncytiotrophoblast. Trophectoderm cells which comprise the wall of the blastocyst can be observed at day 5 post fertilization and differentiate into the syncytiotrophoblast which then covers the blastocyst during initial steps of implantation. Continuously, the syncytiotrophoblast expands henceforward to form the outer surface of the placenta. Human chorionic gonadotrophin (hCG), a major pregnancy hormone, is released in secretory granules formed in the Golgi apparatus [35] and starts to rise in blastocyst culture medium from day 6 post fertilization on [36] and in maternal serum seven days after fertilization. However, at that stage, due to absent vascular connection to the maternal blood circulation, the paracrine action of hCG is limited [37]. In fact, the primary role of hCG is to prolong the endocrine activity of the corpus luteum which produces and secretes progesterone. Moreover, hCG stimulates trophoblast differentiation, uterine angiogenesis and immunotolerance [38]. 

Soon after implantation, vacuoles build within the syncytiotrophoblast, which fuse to bigger lacunae. These lacunae are readily filled with a putative mixture of maternal glandular secretions, maternal blood plasma and blood. The lacunae are separated by trabeculae, villous precursors built of syncytiotrophoblast and filled with continuously proliferating cytotrophoblasts. Some cytotrophoblasts invade as extravillous trophoblasts from the primary villi into the maternal tissues and enter maternal uterine blood vessels (arteries, veins, lymphatics), glands and decidual stroma. Thus, extravillous trophoblasts anchor the placenta, invade and remodel uterine blood vessels to optimize maternal blood flow to and from the placenta [39,40]. The access to the maternal bloodstream facilitates the accession and systemic action of hormones and proteins secreted by the placenta in the maternal circulation. 

The intervillous space is connected with the maternal vessels already very early in pregnancy. Thus, hCG and other relevant substances can be released from the placenta into the maternal circulation via the following path: syncytiotrophoblast–intervillous space–invaded uterine veins–maternal circulation [41,42]. However, in the first trimester, maternal blood flow through the intervillous space is restricted by extravillous trophoblast plugs. Trophoblast plugs result from extravillous trophoblast invasion into the uterine spiral arteries which limit blood flow through the spiral arteries towards the placenta in the first trimester [43]. The hormonal release from the placenta into the maternal circulation goes in line with a shift from low to high maternal blood flow, through the intervillous space: low in the first trimester in presence of trophoblast plugs, high after disintegration of plugs at the end of the first trimester [43]. This change in blood flow thus represents a further mechanism to increase systemic hormone and protein release of the placenta. 

Therefore, the combination of endocrine activity of the trophoblast, placental growth, and consequently, size, as well as the developmentally regulated access to the maternal circulation, modulate the maternal levels of placenta derived hormones and bioactive molecules. 

## 6. Placental Hormones: Role in Glucose Metabolism and Sex Dimorphism

**Figure 2:**
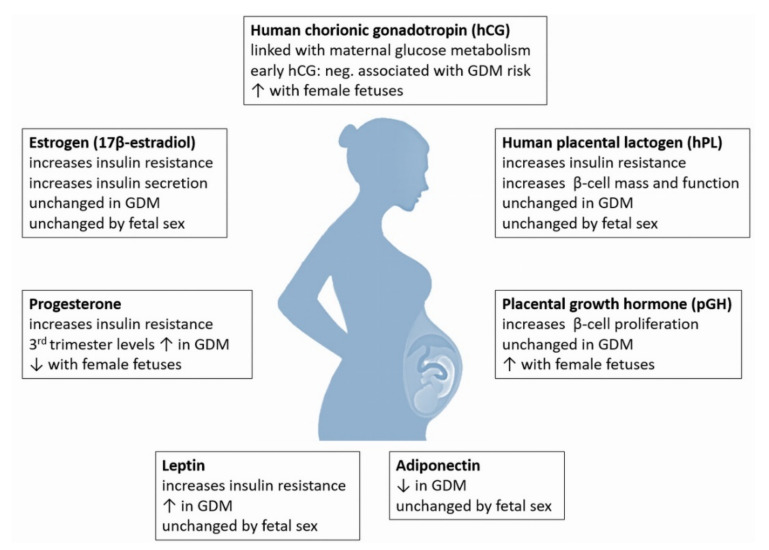
The placenta is an endocrine organ which produces several metabolic proteins (leptin, adiponectin), peptide hormones (hCG, hPL, PGH) and steroid hormones (progesterone, estrogens) which have significant influence on maternal glucose metabolism and its adaptation throughout pregnancy (modified from [12]).

Studies in rodents have first indicated that pregnancy-related hormones are able to influence maternal β-cell function and the peripheral tissue sensitivity to insulin. However, whether or to what extent the proposed mechanisms can be transferred from rodents to humans is still being controversially discussed. The following chapter outlines important placenta-derived hormones and bioactive proteins, their potential role in increasing maternal insulin resistance and β-cell function, and whether they are altered by GDM or by fetal sex. An overview of is shown in Figure 2. 

### 6.1. Early hCG Is Negatively Associated with the Risk for GDM Diagnosis

hCG is a glycoprotein hormone considered primarily as a product of the syncytiotrophoblast, although it can also be secreted by several normal non-placental tissues and trophoblastic or non-trophoblastic neoplasms [44,45]. The standard hCG molecule is released as a heterodimer, consisting of an α- and a β-subunit polypeptide chain (α-hCG, β-hCG). hCG can be detected in maternal serum as early as seven days after fertilization and eight days after ovulation. During the in vitro fertilization procedure, intact hCG molecules are synthesized by the eight-cell embryo and appear between two and eight days after fertilization in the embryo’s culture medium [36]. After implantation of the blastocyst, hCG concentration increases exponentially in the first trimester, doubling approximately every 48h, and peaking between 11 and 13 weeks of gestation. Thereafter, at 20 weeks of gestation, hCG declines to 80% and remains at this concentration until term. hCG has long been considered solely for its luteotrophic effect, acting on luteal cells of the maternal corpus luteum to maintain progesterone production in the ovary, and hence, ensuring maintenance of the highly differentiated endometrium (i.e., decidua). More recently, many other important functions have been described for this versatile hormone. During early stages of pregnancy, hCG regulates hemochorial placentation, which includes the regulation of uterine, fetal, and placental growth, as well as the protection of the pregnancy from myometrial contraction and from immune rejection [46].

Despite some controversy, perhaps due to different composition of patient cohorts and/or heterogeneous diagnostic criteria for GDM, an increasing body of evidence suggests a link of maternal serum hCG and maternal glucose metabolism. Accordingly, hCG and free β-hCG in early pregnancy is negatively associated with GDM risk [47,48,49,50]. This association has been confirmed in GDM-derived primary trophoblasts, which showed significantly decreased expression of genes associated with differentiation, including impaired synthesis of the hCG-beta subunit [51]. Moreover, serum β-hCG is negatively correlated with the homeostasis model assessment of insulin resistance index (HOMA-IR) [52]. Glucose per se may not directly affect placental hCG synthesis, since dual perfusion of isolated human placental cotyledons with medium supplemented with increased glucose had no effect on placental hCG secretion [53]. However, physiological concentrations of insulin inhibit hCG secretion in first trimester placenta explants [54] and the release of β-hCG from trophoblast cell line JAR, when cultured in absence of fetal calf serum [55]. Another recently suggested signaling route through which hCG may be associated with GDM is the thyrotropin (TSH) receptor axis [47]. By its weak affinity for the TSH receptor, hCG has been suggested to stimulate the thyroid gland and increase serum free thyroxine, which promotes insulin secretion and maintains glucose homeostasis. 

The finding that maternal hCG is elevated in pregnancies with female fetuses dates back to the 1980s [56]. Large cohort studies based on in vitro fertilization (IVF) interventions and first trimester screening for Down syndrome have confirmed these data and quantified the relationships in more detail: As early as day 14 post fertilization, maternal hCG levels are higher by 18% in pregnancies with female embryos [57,58]. During the first trimester, different studies showed between 6% and 15% [59,60,61] higher maternal hCG levels in pregnancies carrying female fetuses. A large study analyzing samples from 1.1 million women revealed 11% higher hCG levels in the first trimester and 8% higher levels in the second trimester in case of a female fetus [62]. hCG levels remain higher in pregnancies with girls during the third trimester [63] and at birth [64]. The observation that GDM is associated with lower hCG levels and the notion that hCG can act anti-diabetogenic via the thyroid hormone regulatory pathway fit well with the finding that pregnancies with girls show higher maternal hCG levels and thus might have a reduced GDM risk. 

### 6.2. Placental Lactogen Increases Maternal β-Cell Mass and Function

Based on structural similarities, human placental lactogen (hPL) is a member of the somatotropin family, which also includes growth hormone (GH), placental growth hormone (pGH, encoded by *GH2*, alias *GH-V*), and prolactin (PRL) [65]. hPL, also known as human chorionic somatomammotropin, is detectable in both umbilical cord blood and maternal blood, starting at sixth week of gestation. In the second half of pregnancy, its concentrations increase 10-fold until finally reaching a plateau in the last four weeks of gestation. With progressing placentation, maternal circulating levels of prolactin (PRL), released from the maternal anterior pituitary, decline rendering hPL the dominant lactogenic hormone. hPL predominantly acts through prolactin receptors (PRLR) and, albeit with a much lower binding affinity, also through growth hormone receptors [66]. Like other placenta-derived hormones, hPL could promote the state of systemic insulin resistance and subsequently adapt the maternal glucose and fat metabolism to ensure the continuous supply of glucose and amino acids to the growing fetus [67].

hPL also acts via the PRLR on maternal β-cells to increase β-cell mass and function, promoting their adaptations to increased insulin requirements during normal pregnancy. The relevance of this hPL—PRLR interaction in pregnancy becomes apparent by several single nucleotide polymorphisms of the *PRLR* gene, which are associated with an increased risk for GDM [68]. A direct effect of hPL on β-cells has previously been confirmed in vitro in adult human islets. Cultured in the presence of lactogenic hormones, islets responded with increased glucose-induced insulin secretion [69]. However, a potential influence of hPL on the development of GDM is still not well established. While pre-gestational diabetes has been suggested to be associated with increased blood levels of hPL [70], differences in hPL blood levels between patients with GDM and controls are still at debate. Two studies with a relatively large number of enrolled patients showed no differences in hPL levels between GDM cases and controls [70,71]. Moreover, although higher levels of hPL were found in umbilical cord blood of female fetuses, no sex difference was identified in maternal blood [72]. 

### 6.3. Placental Growth Hormone Is Not Altered in GDM but Can Be Associated with Macrosomia

As another member of the somatotropin family, placental growth hormone (pGH) shares approximately 85% homology with hPL, and close similarity with human growth hormone (hGH, pituitary GH), which is predominantly released from somatotroph cells of the anterior pituitary gland. pGH is expressed in the syncytiotrophoblast and extravillous trophoblasts, and is secreted by the placenta into the maternal circulation in a non-pulsatile fashion. Systemic levels of pGH gradually increase with transition to second trimester and replace hGH towards term of gestation [73]. In contrast to some other placenta-derived hormones, pGH is secreted solely into the maternal, but not into the fetal circulation [74]. pGH is considered a major regulator of maternal insulin-like growth factor I (IGF-I), as maternal total pGH and IGF-I levels significantly correlate and are decreased in pregnancies affected by intrauterine growth retardation [75]. However, since pGH is not detectable in the fetal circulation, impaired fetal growth may rather be a consequence of impaired placenta development and transplacental nutrient transport. Receptors for pGH are expressed on the placenta and argue for autocrine/paracrine mechanisms by pGH [76].

Interestingly, amongst placenta-derived hormones, only pGH secretion can be modulated by glucose, as demonstrated in term villous explants and trophoblast cultures. Treatment with glucose resulted in a dose-dependent reduction of pGH production [77]. This observation is supported by in vivo data, showing that pGH levels decline during oral glucose test in women with GDM [76]. pGH can efficiently signal through PRLR [9] and through the GH receptor (GHR). Both are located on β-cells and their activation stimulates glucose-induced insulin secretion [69,78]. *GHR* mRNA is markedly increased in the pancreas of pregnant rats [79], suggesting a role GH in the stimulation of β-cell proliferation and function. However, a study investigating a small GDM cohort reported that maternal blood pGH levels are not different between GDM cases and controls at twenty weeks of gestation [80]. Interestingly, a sex dimorphism in the maternal pGH levels has been described with higher levels in pregnancies with female fetuses in the third trimester of pregnancy [81]. 

### 6.4. Adiponectin/Leptin Ratio Is Inversely Associated with GDM

Adiponectin and leptin are both adipokines, i.e., adipocyte-secreted hormones, involved in metabolic disease-related vascular dysfunction, including obesity and T2D [82]. While adiponectin and leptin are synthesized mainly by adipose tissue, both are also reported to be released from the placenta and secreted into maternal circulation [3,83,84]. In non-pregnant state, leptin enhances peripheral insulin sensitivity, improves β-cell function [85], and suppresses food intake as a satiety signal [86]. However, pregnancy is associated with leptin insensitivity which suppresses the action of leptin and may even promote insulin resistance and impairment of glucose tolerance [87]. Plasma leptin concentrations are two-fold higher during pregnancy than in the non-gravid state, reaching highest values at 28th week gestation. Substantial evidence suggests that the placenta, rather than maternal adipose tissue, significantly contributes to the rise in maternal leptin levels [87]. In fact, high amounts of leptin mRNA and protein, which is identical to leptin of adipose origin, are produced in the human placenta in early, mid, and late gestation [88]. On cellular level, placental leptin is expressed in the syncytiotrophoblast and fetal vascular endothelial cells. According to in vitro studies using dually perfused human placenta cotyledons, approximately 95% of total placental leptin is released into the maternal circulation [89]. Leptin of either placental or maternal origin can induce production of hCG through binding placental leptin receptors [90]. In this context, it is worth mentioning that adiponectin increases hPL expression in human placenta explants and the trophoblast cell line JEG3 [91], substantiating a fundamental role of adipokines in regulating placenta-derived hormones.

In contrast to leptin, maternal circulating adiponectin levels progressively decline during pregnancy and are even lower in women with GDM [92]. This decrease is proportional to the increase in BMI, insulin resistance and hemodilution, suggesting that hypoadiponectinemia could be involved in the development of the insulin-resistant state observed in late pregnancy. However, adiponectin does not affect basal or glucose-stimulated insulin secretion or basal or fatty acid-induced β-cells apoptosis, although human β-cells express the adiponectin receptors AdipoR1 and AdipoR2 [93,94]. Maternal leptin levels are associated with gestational insulin resistance/sensitivity, assessed by intravenous glucose tolerance test or HOMA-IR in late pregnancy [95,96]. A large number of studies indicate that women with GDM show elevated plasma leptin levels, when compared with controls, while maternal adiponectin levels were lower in women with GDM [70,97,98]. The hyperleptinemia is suggested to interfere with insulin-dependent glucose transport to adipocytes and to suppress insulin secretion in pancreatic β-cells [99,100]. Moreover, placental leptin and cord blood levels are higher in pregnancies with macrosomic newborns than with normal birthweight newborns. This suggests that placenta-derived leptin, secreted into fetal circulation, may be involved in fetal growth and development of macrosomia, which is a major adverse outcome of GDM [97]. 

Whether a sex dependent difference exists in maternal leptin levels seems controversial: A small study analyzing maternal plasma throughout pregnancy in 37 pregnant women revealed higher leptin levels in all trimesters when women were pregnant with girls [101]. However, investigating leptin in first and second trimester blood samples of 582 pregnant women did not identify any sex dependent difference [30]. Angiopoietin levels seem not different between women carrying a boy vs. women carrying a girl [102]. 

### 6.5. Maternal Estrogen and Adipose Estrogen Receptor Expression Are Altered in GDM

In the initial phase of human pregnancy, estrogens are predominantly synthesized by the corpus luteum. At approximately nine weeks of gestation, the placenta, more specifically, the syncytiotrophoblast takes over the production of the three predominant endogenous estrogens, estrone (E1), 17β-estradiol (E2), and estriol (E3) [103]. Once synthesized, E1 and E2 can be further catabolized into estriol E3 and estetrol (E4); conversion of E1 into E2 takes place exclusively in the placenta [104]. In normal pregnancy, estrogen levels increase progressively towards term. However, due to the lack of some key enzymes, placental steroid hormone synthesis critically depends on steroid precursors, delivered by fetal and maternal steroidogenic organs. Accordingly, placental estrogen synthesis requires dehydroepiandrosterone (DHEA) and its sulfated form dehydroepiandrosterone sulfate (DHEA-S), both provided by fetal and maternal adrenal glands. E3 is released in high amounts during pregnancy, although its biological activity is considered very low. Nevertheless, competitive binding of E3 to estrogen receptors may influence the activity of E2, which is involved in regulating angiogenesis, trophoblast differentiation, and invasion [104].

Estrogens are implicated in adaptation of islets to pregnancy, through both, direct and indirect protective effects on β-cells [105]. This assumption is primarily based on observations in rodent models, demonstrating that E2 can induce β-cell mass expansion via estrogen receptor (ER)α signaling by inducing the replication of existing β-cells or the neogenesis of new β-cells. However, it is important to stress that data from animal experiments still need validation in human β-cells. Estrogens and their receptors are discussed to play important roles in regulating body weight and insulin sensitivity, although exact mechanisms of estrogen-mediated adaptation of β-cell response to insulin resistance is unknown. Recently, maternal unconjugated E3 levels above 95th percentile in early second trimester were significantly associated with an increased risk for developing GDM, suggesting that very high maternal serum levels of E3 in early pregnancy may inhibit the interaction of E2 with its receptor, thus promoting insulin resistance and the development of GDM [106]. While maternal E3 is increased, expression of ERα and ERβ in subcutaneous fat is significantly decreased in women with GDM. Moreover, gene expression of leptin (*LEP*) and ERβ is significantly related in subcutaneous adipose depot [107]. Hence, the concept has been proposed that increased leptin expression in maternal visceral adipose depot together with increased proinflammatory cytokines and reduced expressions of estrogen receptors in subcutaneous fat could play a role in the development of GDM. The finding that, in GDM pregnancies, expression of ERα in extravillous trophoblasts and decidua [108] is upregulated suggests that diabetic conditions not only affect estrogen signaling in maternal adipose tissue, but also in the placenta. 

However, data on sex differences in maternal estrogen levels are conflicting. A large cohort of 1343 participants revealed 9% higher levels of estrogen during the first trimester when women were pregnant with girls [109], which was also found by a small study one year later [110]. However, several studies found no sex dependent differences in the first trimester [101,111], or at term of pregnancy [101,111]. 

### 6.6. Progesterone Is Inversely Associated with Glucose and Insulin in Early Pregnancy

Like estrogens, progesterone is a steroid hormone produced by the corpus luteum during very early phases of pregnancy warranting maintenance of the decidua after implantation of the blastocyst. About 6–10 weeks after last menstruation, progesterone productions shifts from the corpus luteum to the placenta, being now its predominant source [112]. The syncytiotrophoblast then progressively secretes progesterone into the maternal circulation. This leads to induced weight gain and fat deposition, mediated by increased food intake and upregulation of adipogenesis as well as fatty acid synthase expression in pre-adipocyte precursor cells [113,114]. Beside its role in the decidua, progesterone is suggested to modulate the maternal immune response and to suppress the inflammatory response. Moreover, progesterone reduces uterine contractility by counteracting the stimulatory activity of prostaglandin and oxytocin [115].

In human pregnancy, progesterone has been attributed to certain diabetogenic functions, which manifest in the last trimester. These functions are related to insulin resistance due to reduced insulin binding, downregulation of GLUT4 (encoded by SLC2A4), diminished glucose transport in skeletal muscle and adipose tissue, as well as insulin-induced gluconeogenesis in the liver [113,116]. Progesterone receptor (PgR) expression has been demonstrated by immunohistochemistry in human pancreatic islets [117], but direct effects of progesterone on insulin secretion and islet cell proliferation are still a matter of controversial discussion. However, a prospective and longitudinal study recently showed that progesterone levels are significantly lower in GDM pregnancies than controls at weeks 10–14, and significantly and inversely associate with glucose, insulin, and C-peptide levels [118]. At that time of gestation, maternal progesterone levels seem not to differ between pregnancies with male vs. female fetuses [110,111]. Another study found significantly higher levels of progesterone in patients with GDM compared with matched controls, when sampled between gestational week 25 and 29 [119]. Importantly, prophylactic supplementation with the synthetic gestagen 17α-hydroxyprogesterone caproate for prevention of recurrent preterm delivery has been related to an increased incidence of GDM and warrants earlier GDM screening in this particular group of patients [120]. In line with the lower GDM risk in pregnancies carrying girls, two cohort studies with 335 and 142 participants revealed reduced maternal progesterone levels in pregnancies with female fetuses in second and third trimester [121,122]. 

## 7. Hormonal and Molecular Causes Underlying Fetal and Placental Sexual Dimorphisms

Sexual dimorphism presents very early in pregnancy: Increased growth rate of male fetuses is observed already at the stage of blastula in mice and cattle [123]. In humans, this early dimorphism is likely to exist as well, and a growth difference was observed at the 12th week after conception [124]. In contrast to the increased growth of male fetuses, male placentas are smaller, and therefore seem to be more efficient vs. female placentas, postulating less reserve capacity when facing a challenge [125]. Such early sex differences in placental function are attributed to sex chromosomes, as differential gene expression occurs very early on and precedes the production of fetal sex hormones, which can also affect the placenta. 

In fact, embryonic gonads of males and females are indistinguishable until 6 weeks post conception. Testes development is induced and regulated by genes on the Y chromosome, including *SRY* (Sex Determining Region Y) [126,127]. Testosterone production starts at week 9 [128] and rises until week 16. Then, levels slightly fall again [129]. Concordantly, the amniotic fluid testosterone levels are higher in pregnancies with boys in the early second trimester [130], and levels in the fetal circulation remain higher in boys until birth [131]. Such distinct fetal testosterone levels in pregnancies with boys could potentially have effects on placental hormone production. However, levels in maternal circulation seem to be unaffected by fetal sex [109,132]. 

Fetal ovaries start to differentiate even later than testes, between weeks 11–12. As the placenta produces high amounts of estrogen, fetal estrogen does not remarkably contribute to the total levels, and a large cohort study revealed no difference in umbilical cord blood estrogen in girls vs. boys [133]. Similarly, maternal levels do not seem to differ either (cf above). 

Early in development, sex dependent gene expression may be due to genetic and epigenetic differences. Later, fetal sex hormones, foremost testosterone, contribute to the sex dimorphisms. Several mechanisms are proposed on how sex chromosomes set the first milestone of sexual dimorphism in terms of differential gene expression (reviewed in [134]): (a) Female-biased expression of X chromosome genes; (b) Escape from X-chromosome inactivation; (c) Mosaic X chromosome Inactivation; and (d) X-chromosome dosage in female fetuses and (e) Y chromosome gene expression in male fetuses, including *SRY*. Thus, the distinct expression of sex chromosome-linked genes leads to differences in autosomal gene expression [135,136,137]. 

In combination with effects of sex hormones and processes regulated by them, these mechanisms yield the final sex-specific placental phenotypes that in turn, show distinct responses to maternal environmental stimuli [138,139]. The influence of maternal environment on placental function is well documented from conception to birth and epigenetic mechanisms, i.e., DNA methylation and regulation by microRNAs seem to be a major contributor. These mechanisms add another level to the sexual dimorphism of the human placenta as they are also modulated by fetal sex [140,141,142]. 

## 8. Conclusions

In this review, we have highlighted the role of placental hormones in the adaptation of maternal glucose metabolism and elaborated the role of fetal sex in this regulation. Both, in the establishment of physiological pregnancy-associated insulin resistance and in supporting the balancing increase in β-cell mass and function, placenta derived hormones and metabolic proteins play a central role. Although the cohort size of studies has tended to increase in recent years, results regarding sex differences in glucose metabolism and placental hormones in the maternal circulation are not always clear.

Further studies are needed, optimally examining parameters of glucose metabolism in parallel with placental hormones, to fully elucidate the fascinating relationships between fetal regulation of the maternal organism via the placenta. 

## AuthorContributions

Conceptualization, U.H.; C.S.; S.S.; G.M.; S.C.; E.J.-K.; M.G. and U.H. have written on the manuscript and read and agreed to the published version of the manuscript. All authors have read and agreed to the published version of the manuscript.
